# Postpartum-Isolated Native Pulmonic Valve Methicillin-Resistant *Staphylococcus aureus* Infective Endocarditis Complicated by Pelvic Abscess, Clavicle Osteomyelitis, and Polyarticular Septic Arthritis

**DOI:** 10.1155/2020/8850340

**Published:** 2020-11-06

**Authors:** Daniela Navarrete, David Hannibal, Sachin M. Patil, Tarang Pankaj Patel, William Roland

**Affiliations:** ^1^Department of Medicine and Pediatrics, PGY4 Resident, University of Missouri Hospital and Clinic, 1 Hospital Dr, Columbia, MO 65212, USA; ^2^University of Missouri School of Medicine, MS 4, University of Missouri Hospital and Clinic, 1 Hospital Dr, Columbia, MO 65212, USA; ^3^Department of Medicine, Division of Infectious Disease, University of Missouri Hospital and Clinic, 1 Hospital Dr, Columbia, MO 65212, USA; ^4^Department of Medicine, Division of Pulmonary, Critical Care and Environmental Medicine, University of Missouri Hospital and Clinic, 1 Hospital Dr, Columbia, MO 65212, USA

## Abstract

Isolated native pulmonic valve infective endocarditis (IE) is a rare occurrence. The most commonly involved valves in injection drug users are the tricuspid valve followed by mitral and then aortic valves. Most reported cases of methicillin-resistant *Staphylococcus aureus* (MRSA) IE involve multiple valves. Isolated involvement of the pulmonic valve in IE is infrequent, especially in intravenous drug users or patients with indwelling catheters, prosthetic valves, or implantable cardiac devices. Here, we report a young postpartum female patient with isolated native pulmonic valve MRSA IE with MRSA bacteremia and history of active injection drug use. A PubMed literature review revealed a single described prior case report in a postpartum female. The patient's clinical course was complicated by a large native pulmonic valve vegetation, septic pulmonary emboli, pelvic abscess, polyarticular septic arthritis, and clavicular osteomyelitis. The patient underwent bioprosthetic pulmonic valve replacement and finished six weeks of intravenous vancomycin for complete recovery.

## 1. Introduction

The opioid crisis has become an increasingly prevalent public health and medical issue in the United States over the past 15 years [1]. In 2016, nearly 43,000 people died from opioid-related deaths, which is expected to rise to almost 100,000 per year [1]. Opioid addiction can be secondary to prescription opioids or illicit drugs. IE among injection drug users is approximately 2% to 5% per year and responsible for up to 20% of hospital admissions with a mortality of 10% [2]. The opioid crisis places an economic health burden, and therefore, addiction treatment and emphasis on addiction medicine are essential.

IE in injection drug use most commonly involves the tricuspid valve followed by the aortic and mitral valves. Pulmonic valve IE is an infrequent entity, and most of these cases do not have severe complications such as sepsis from septic emboli [3]. We present an injection drug user with methicillin-resistant *Staphylococcus aureus* (MRSA) bacteremia and pulmonic valve IE complicated by septic emboli to lungs, polyarticular septic arthritis. There is higher mortality in patients with both IE and septic arthritis [4], and injection drug users are at risk. Our case demonstrates that in injection drug users with bacteremia and IE, a thorough evaluation of different concurrent infections should be done. Musculoskeletal complaints should prompt further imaging.

## 2. Case Presentation

A 35-year-old white female presented to the emergency room for severe midsternal chest pain and left shoulder pain that started the previous day. She had a recent vaginal delivery at home approximately ten weeks before this presentation and had ongoing intravenous drug use. Since the birth at home, she had lower abdominal pain but failed to seek medical attention. She also reported two to four weeks of generalized malaise, fever, and chills. She was febrile at the emergency room, with tachycardia of 105 beats per minute, normal blood pressure, and oxygen saturation >90% on room air. Based on her clinical history and physical examination findings such as tachycardia and severe lower abdominal tenderness, acute pulmonary embolism was a concern. She underwent a computed tomography (CT) scan of the chest, abdomen, and pelvis for further evaluation. Imaging revealed multiple small pulmonary emboli and findings concerning septic arthritis of the pubic symphysis and edema of the left clavicle. Based on her clinical presentation and risk factors, IE was suspected. Therefore, she was transferred to our hospital for further management.

**Figure 1:**
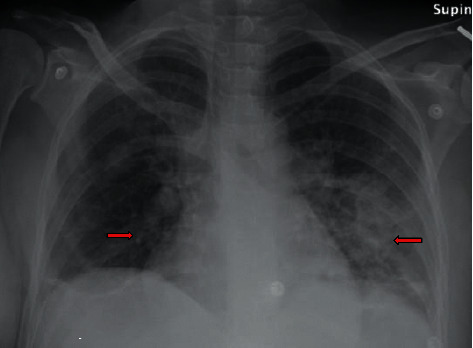
Chest X-ray revealed diffuse bilateral lower lobe predominant airspace opacities with nodularity.

**Figure 2:**
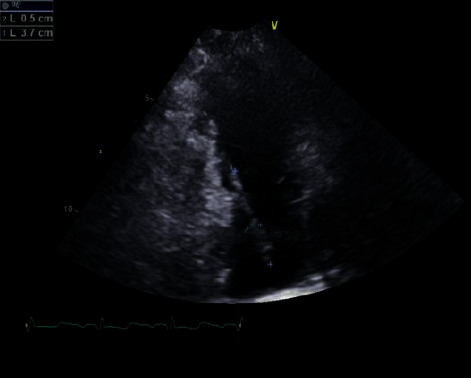
Transthoracic echocardiogram revealed extensive mobile 3.7 × 0.5 cm vegetation in the pulmonary valve with moderate to severe pulmonic regurgitation mobile 3.7 × 0.5 cm vegetation in the pulmonary valve with moderate.

On arrival at our facility, the patient had a fever of 38.8ºcelsius, tachycardia of 125 beats per minute, blood pressure of129/79 mmHg, and oxygen saturation of 93% on room air. Labs revealed leukocytosis of 19,000/mL with neutrophil predominance and normal renal and hepatic functions. Labs were significant for hypoalbuminemia and elevated inflammatory markers, including ESR (Erythrocyte sedimentation rate) of 100 mm/Hr and CRP (C-reactive protein) of 27 mg/dL. Her urine drug screen was positive for amphetamines and opiates, and she admitted to the daily use of heroin and methamphetamine. On physical examination, she was alert and oriented but appeared to be in painful distress, expressing significant abdominal and left shoulder pain. Lungs were clear on auscultation, whereas on the cardiac exam, a diastolic murmur in the pulmonary area was heard. She had severe suprapubic area tenderness on palpation. The left shoulder limited range of motion with no spine tenderness was appreciated on musculoskeletal examination. Left antecubital and buttock areas showed the presence of multiple needle marks with no signs of infection. A portable chest X-ray disclosed diffuse lung opacities suspicious of septic emboli (Figure 1). Empirically intravenous (IV) vancomycin and ceftriaxone were initiated for suspected IE treatment. The transthoracic echocardiogram (TTE) demonstrated an extensive mobile 3.7 × 0.5 cm vegetation on the pulmonary valve with moderate to severe pulmonic regurgitation (Figure 2). The patient refused a transesophageal echocardiogram (TEE) despite multiple requests. The cardiothoracic surgery team review of the TTE was negative for any left-sided IE stigmata.

**Figure 3:**
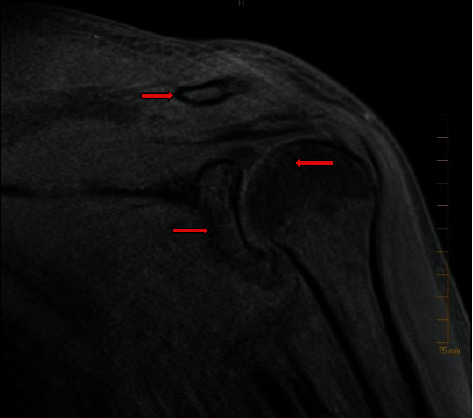
MRI left shoulder revealed acromioclavicular joint synovitis with periarticular soft tissue edema consistent with septic arthritis and distal clavicle marrow edema suggestive of osteomyelitis. A subcentimeter abscess in the subacromial bursa was also noted.

**Figure 4:**
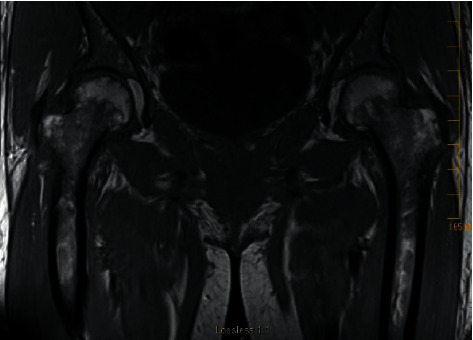
MRI pelvis revealed peripherally enhancing fluid collection extending into the adjacent soft tissues from the pubic symphysis consistent with septic arthritis. Left pubic body erosion without confluent marrow replacement indicates osteomyelitis. It also shows bilateral hip joint effusions with nonspecific left hip synovitis.

Magnetic resonance imaging (MRI) of the left shoulder revealed acromioclavicular joint septic arthritis and abnormal marrow edema in the distal clavicle concerning for osteomyelitis (Figure 3). A subcentimeter abscess in the region of subacromial bursa was also seen (Figure 3). Pelvis MRI showed peripherally enhancing fluid collection from pubic symphysis compatible with septic arthritis and erosion in the left pubic body without confluent marrow replacement to indicate osteomyelitis and bilateral hip joint effusions (Figure 4). Blood cultures returned positive for MRSA within 12 hours, and they remained positive from the first day of hospitalization until day 7. The mean inhibitory concentration of vancomycin for MRSA was 1. The positive blood cultures resulted in the de-escalation of antibiotics to vancomycin alone.

The patient underwent irrigation and debridement of the pelvic abscess and left shoulder joint. The cultures from the fluid aspiration of the left acromioclavicular joint and pubic symphysis grew MRSA confirming septic arthritis. On the 13th day of hospitalization, she underwent bioprosthetic pulmonic valve replacement and pulmonary artery reconstruction with autologous pericardial patch placement. Intraoperative echocardiogram did not reveal any left-sided IE stigmata. The special stain for fungal organisms was negative. Cultures from the valve were not sent due to positive blood and tissue cultures from the joint aspirations. Histopathology of the pulmonic valve vegetation revealed a partially necrotic cardiac valve with septic vegetations, numerous Gram-positive cocci in clusters, and pairs. After one week following cardiac valve surgery, she had dental extraction of the remaining upper teeth to optimize dental hygiene. After valve replacement, she was started on warfarin with an international normalized ratio goal of 2-3.

Due to the history of active ongoing intravenous drug abuse, outpatient parenteral antibiotics were deferred. Instead, the patient received intravenous antibiotics, inpatient, until completion of her antibiotic course. She completed the IE treatment with six weeks of vancomycin. Inpatient pharmacy managed the vancomycin dosing based on the trough levels. Vancomycin dosing was three times a day, and the average dose ranged from 750 mg to 1250 mg over six weeks. Her mean vancomycin trough levels ranged from 15 mcg/mL to 18 mcg/mL. Upon completing antibiotic therapy, the ESR had decreased to 32 mm/Hr and CRP decreased to 1.32 mg/dL. Before being discharged home, she underwent an evaluation by psychiatry for suboxone treatment. She was discharged home in a stable condition. Unfortunately, she was lost to the outpatient clinic follow-up and repeat imaging to ensure that the infection resolution could not be obtained.

## 3. Discussion

IE is an infection of the endocardium or a prosthetic valve surface associated with a one-year mortality rate of up to 40% and an in-hospital mortality rate of about 20% [5]. These alarming statistics make this an important topic in healthcare. The outcomes have not improved substantially over the past 25 years, despite many advances in medical and surgical treatment [6]. Fortunately, IE is a rare disease with an incidence of 15 per 100,000 people in the United States in 2011 [7]. However, with the rising opioid crisis and injection drug use as a contributing fuel, IE cases will increase and place a burden on healthcare costs due to the need for hospitalization. An emphasis on the field of addiction medicine for management is one of the most vital aspects of overcoming the burden.

Our case involves the uncommon occurrence of pulmonic valve IE in a patient with intravenous drug use. In injection drug users, 60% of cases of IE occur on the tricuspid valve, and in about 40% of cases, the left heart valves alone are affected. The pulmonic valve is rarely involved, and this may be due to the low-pressure gradient and low wear and tear of this valve [8]. In all patient populations with infective endocarditis, pulmonary valve infections account for ≤2% of cases [9].

Most cases of pulmonary valve IE occur in children with congenital heart disease [3]. As a result of pulmonic valve IE being so rare, the diagnosis can be easily missed. A significant number of patients with pulmonic valve IE have pulmonary symptoms such as dyspnea, chest pain, and cough [10]. There is often a delay in diagnosis due to the lack of typical signs and symptoms seen with mitral or aortic endocarditis [3]. Modified Duke criteria facilitate pulmonic valve IE determination, and further evaluation includes a thorough physical exam, TEE, and blood cultures [11, 12].

In our patient, the pulmonary valve vegetation was visible on a TTE due to its large size. Although right-sided IE has a better prognosis than left-sided, it should not be neglected as the potential pulmonary emboli and IE complications seen in our patient can be fatal. Therefore, it is essential to pursue a TEE in patients with suspected infection and significant risk factors for IE, like injection drug use. IE and *Staphylococcus aureus* (*S*. *aureus*) bacteremia can become further complicated by infectious foci at any site. Our patient had a pelvic abscess, clavicular osteomyelitis, and polyarticular septic arthritis. It is rare for patients with IE to have septic arthritis with rates ranging from 5 to 31% [4]. *S*. *aureus* is one of the most commonly isolated organisms from synovial fluid in these patients, as was seen in our case, and intravenous drug use is a risk factor for both IE and septic arthritis [4].

Treatment of IE is 4–8 weeks of intravenous antibiotics after the offending bacteria is identified [13]. The offending bacteria are often *S*. *aureus*, coagulase-negative staphylococci, or Group B streptococci [3]. Surgery is an option in patients with progressive heart failure, an infection resistant to antibiotics, complications such as abscess formation or recurrent pulmonary embolism, or vegetation greater than 2 cm [12, 13]. Our patient had pulmonary valve replacement due to the vegetation size and for source control.

Our case of pulmonic valve IE involved a patient during her postpartum period. IE is rare and life-threatening during pregnancy [14]. The calculated maternal mortality is 11.1%, and the fetal mortality rate is 14.3% [14]. The mortality risk has improved most likely due to maternal-fetal monitoring, new surgical techniques, and nontoxic and effective antimicrobial agents [14]. Pregnant patients' risk factors, microbiology, and IE complications are similar to those of nonpregnant patients. During pregnancy, risk factors include injection drug use, congenital heart disease, and less commonly rheumatic heart disease [14]. Rheumatic heart disease has decreased, and the number of cases due to injection drug use has increased markedly. Streptococcal species are the most common pathogen in pregnant patients, followed by staphylococcal species [14]. However, right-sided infective endocarditis is more likely to be staphylococcal species [14].

On review of the PubMed literature, ours is the second case of isolated native pulmonic valve MRSA IE. Interestingly, the first reported case also was seen in a 19-year-old postpartum female [15]. Pulmonary, central nervous system, and systemic emboli are common complications [14]. The treatment for IE in pregnant women most commonly consists of a multidrug antibiotic regimen [14]. Selected patients may need surgical intervention and antibiotics. The surgical options include a valve replacement or valvuloplasty [14].

## 4. Conclusion

This case demonstrates IE's unique presentation due to isolated native pulmonic valve disease with concurrent septic arthritis and osteomyelitis during the postpartum period. Our patient presented with polyarticular septic arthritis and left clavicular osteomyelitis in the setting of right-sided IE. This case emphasizes the importance of recognizing the possibility of pulmonary septic emboli and other infectious foci in pulmonic valve endocarditis, especially in high-risk populations such as injection drug users.

## Data Availability

Previously reported data were used to support this case report and are available within the manuscript. These prior studies are cited at relevant places within the text as references [1–15].
